# Severity of Acute Kawasaki Disease Can Be Predicted by Evaluating the Body Temperature at the Completion of an Initial Immunoglobulin Treatment

**DOI:** 10.3390/jcm13226985

**Published:** 2024-11-20

**Authors:** Shintaro Kishimoto, Tamotsu Fujimoto, Kenji Ihara

**Affiliations:** 1 Department of Pediatrics, Oita University Faculty of Medicine, 1-1 Idaigaoka, Hasama, Yufu 879-5503, Japan; skishimoto0131@oita-u.ac.jp; 2 Department of Pediatrics, Oita Children’s Hospital, 837 Katashima, Oita 870-0943, Japan; fujimoto@oita-kodomo.jp

**Keywords:** Kawasaki disease, initial intravenous immunoglobulin, body temperature, early prediction, coronary artery abnormalities

## Abstract

**Objective**: We aimed to determine whether the severity of acute Kawasaki disease (KD) can be predicted based on whether a patient remains febrile or becomes afebrile immediately after the completion of initial immunoglobulin treatment (IVIG). **Methods**: This retrospective cohort study at a single institution involved 306 patients with KD. They were categorized into four groups according to their fever status at two specific time points (end of the initial IVIG treatment and 24–36 h later): Group F-F, patients who remained febrile at both time points; Group F-AF, patients who were febrile at the end of the initial IVIG treatment but became afebrile 24–36 h later; Group AF-F, patients who were afebrile at the end of the initial IVIG treatment but became febrile 24–36 h later; and Group AF-AF, patients who remained afebrile at both time points. The clinical characteristics of the groups were compared. **Results**: Group F-F (n = 38) showed a significantly higher incidence of CAAs compared to Group AF-F (n = 37), 55.3% vs. 0.0% (*p* < 0.0001), although both groups were classified as resistant to the initial IVIG. **Conclusions**: In Japanese patients with acute KD, the presence or absence of fever at the completion of initial IVIG treatment may serve as an early predictor of the occurrence of CAAs. An earlier secondary treatment may be warranted for patients who are in a febrile state immediately after the completion of the initial IVIG treatment. Future research should include a prospective cohort study with a larger number of KD cases across multiple institutions to analyze the effects of other contributing factors related to CAL formation.

## 1. Introduction

Kawasaki disease (KD) is the most common cause of acquired heart disease in children in developed countries and is characterized by systemic vasculitis that causes complications such as coronary artery abnormalities (CAAs) [1,2,3]. The mechanism of coronary artery involvement in KD could be derived from abnormal inflammatory processes [2]. Recent studies have elucidated that the immune system in KD patients becomes excessively activated, triggering inflammatory responses in the vascular endothelium. No infectious causes have been identified as potential underlying etiologies [4,5], while unknown stimulus factors trigger an inflammatory cascade with the activation of both the innate and adaptive arms of the immune system. The innate immune system may be activated by either pathogen-associated molecular patterns (PAMPs) or damage-associated molecular patterns (DAMPs) [6,7]. The NLRP3 inflammasome recognizes these abnormal molecular patterns and activates a signaling cascade, resulting in the release of several pro-inflammatory cytokines. The most studied cytokines in KD include IL-1, IL-18, IL-6, TNF-a, IFN-gamma, and IL-8 [8]. A significant activation of the adaptive response is also important as revealed by increased numbers of circulating pro-inflammatory and regulatory T cells in the acute phase of KD [9]. As a result, cytokines storm with the activation of T cells by the adaptive immune system, and innate immune cells synergistically contribute to inflammation, leading to endothelial cell damage in the coronary arteries.

The standard initial treatment for patients with KD consists of intravenous immunoglobulin (IVIG) and oral acetylsalicylic acid (ASA) [1,2,3]. IVIG, as a broad-spectrum immune modulator, helps to suppress this abnormal immune response. It reduces the levels of pro-inflammatory cytokines, inhibits the formation of immune complexes, and blocks the adhesion of white blood cells to the endothelium, preventing vascular damage and mitigating the development of CAL [1,10,11]. ASA is a key therapeutic agent primarily due to its anti-inflammatory properties. It protects vascular endothelial cells by inhibiting the platelet-aggregating effects of thromboxane A2. The efficacy of low-dose aspirin may be adequate for the initial treatment of KD and can also reduce the risk of complications caused by high-dose ASA [12,13,14,15,16].

Histopathologic studies of patients with KD revealed the following timeline after disease onset: on days 6–8, the media of affected coronary arteries exhibited an edematous dissociation of smooth muscle cells; on days 8–10, numerous inflammatory cells had infiltrated all layers of the affected coronary arterial wall; and on days 10–12, the dilation of affected coronary arteries had begun [17,18]. Therefore, the Japanese guidelines for acute KD recommend the treatment should aim to be successful by the 9th day of illness [19].

Approximately 20% of patients with acute KD, however, require additional treatment for persistent or recurrent fever after the initial IVIG treatment. The Japanese guidelines for acute KD recommend the initiation of second-line treatment if the patient remains febrile (axillary temperature ≥ 37.5 °C) 24–36 h after completing the initial IVIG. However, making an earlier decision on a second-line treatment can help reduce inflammatory damage to the coronary arterial walls. We investigated whether the severity of acute KD could be estimated earlier based on the presence or absence of fever at the time of completing the initial IVIG treatment.

## 2. Materials and Methods

### 2.1. Materials

This single-institution retrospective cohort study aimed to investigate whether the severity of acute KD can be estimated at the completion of an initial IVIG treatment. From April 2017 to December 2021, 309 consecutive patients with acute KD were admitted to Oita Children’s Hospital. The diagnostic criteria for KD were based on the revised 5th edition of the Japanese Kawasaki Disease Society’s diagnostic guide for Kawasaki disease [20] used until April 2019 and the revised 6th edition [21] used from May 2019. In these patients, we collected the following information from medical records: age, sex, day of illness at initiation of the initial IVIG, blood laboratory findings just before the initial IVIG treatment (neutrophil ratio, platelet count, aspartate transaminase (AST), sodium (Na), C-reactive protein (CRP)), echocardiographic findings of the coronary artery, course of treatment, and febrile status at the completion of the initial IVIG treatment and 24–36 h after completing the initial IVIG treatment. Febrile state was defined as an axillary temperature of 37.5 °C or higher. The selection of blood laboratory findings before the initial IVIG treatment was based on indicators that may reflect the severity of vasculitis in KD, as reported in a previous study [22]. All patients were treated uniformly with antipyretics. We did not have sufficient information on acetaminophen use before and after the initial IVIG treatment, and the data were not included in our survey items. No patient had received systemic corticosteroid treatment at least within 3 months before the initial IVIG treatment. Three patients who first visited our hospital on day ≥10 of illness were excluded from the study. Thus, a total of 306 patients with acute KD were enrolled in our study.

### 2.2. Ethics

This study was conducted in accordance with the Declaration of Helsinki. The study protocol was approved by the Ethics Committee of the Oita University Faculty of Medicine (approval number 2490) and Oita Children’s Hospital (approval number 2202). An informed consent for this study was obtained from the patients or their parents during a follow-up period that extended over several years for most patients. Alternatively, we have adopted an opt-out system on the hospital website.

### 2.3. Treatment Strategy for Acute Kawasaki Disease

All enrolled patients were treated according to the Japanese Guidelines for acute KD [19]. All patients were initially treated with IVIG (2 g/kg) and oral ASA (30 mg/kg/day). IVIG was administered for a maximum period of 24 h. If the patient remained febrile 24–36 h after completing the initial IVIG treatment, a second-line treatment (i.e., 2nd IVIG treatment) was performed. Patients who were also resistant to 2nd IVIG treatment received an additional treatment with infliximab, cyclosporine, or plasma exchange until they became afebrile without any recurrent fever. ASA was reduced to 5 mg/kg/day at 72 h after defervescence (axillary temperature < 37.5 °C) and was continued for at least 8 weeks after the onset of KD. Acetaminophen was administered at intervals of at least 6 h when the patient’s temperature reached 38.5 °C or higher.

### 2.4. Evaluation of Coronary Artery Abnormalities

All patients underwent echocardiography to measure the diameters of the proximal right coronary artery (RCA), left main trunk (LMT), and proximal left anterior descending artery (LAD). The diameters of the RCA, LMT, and LAD were calculated as z-scores using the Japan z-score calculator [23]. Echocardiography was performed immediately before the initial IVIG and at 1, 2, and 4 weeks after the onset of KD. The largest z-score among the three branches was defined as the maximum z-score (Zmax), and CAAs were defined as Zmax ≥ 2.0.

### 2.5. Grouping by Febrile Status Just After Completing Initial IVIG and 24–36 h Later

**Figure 1:**
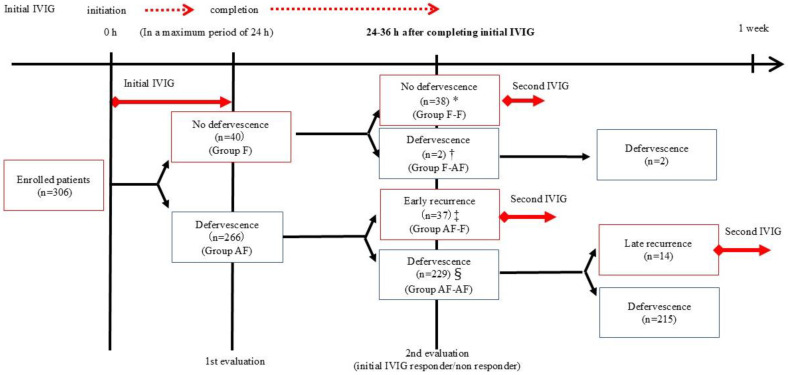
Categorization of patients according to febrile status at the completion of the initial IVIG treatment and 24–36 h later. * Patients who remained febrile both at the completion of the initial IVIG treatment and 24–36 h after the completion of the initial IVIG treatment (Group F-F). † Patients who were febrile at the completion of the initial IVIG treatment but who became afebrile–24–36 h after the completion of the initial IVIG treatment (Group F-AF). ‡ Patients who were afebrile at the completion of the initial IVIG treatment but who became febrile–24–36 h after the completion of the initial IVIG treatment (Group AF-F). § Patients who remained afebrile both at the completion of the initial IVIG treatment and 24–36 h after the completion of the initial IVIG treatment (Group AF-AF).

A Febrile status was defined as an axillary temperature of 37.5 °C or higher. All enrolled patients were divided into the following four groups based on their febrile status at two time points (immediately after the completion of the initial IVIG treatment and again 24–36 h after completing the initial IVIG treatment) (Figure 1): Group F-F, patients remained febrile at both time points (n = 38); Group F-AF, patients were febrile at the end of the initial IVIG treatment but became afebrile 24–36 h later (n = 2); Group AF-F, patients were afebrile at the end of the initial IVIG treatment but were febrile 24–36 h later (n = 37); and Group AF-AF, patients were afebrile at both time points (n = 229).

### 2.6. Statistical Analysis

All statistical analyses were performed using JMP version 17 (SAS Institute Inc. Cary, NC, USA). Categorical variables are expressed as numerical values (%), while continuous variables are expressed as medians and interquartile ranges (IQRs). Missing data were excluded from the statistical analysis. Group F-AF was excluded from the statistical analysis owing to the small sample size (n = 2). *p* values < 0.05 were considered to indicate statistical significance.

To determine whether the severity of acute KD could be predicted at the completion of the initial IVIG, we compared the clinical characteristics, blood laboratory findings before the initial IVIG treatment, and echocardiographic findings of the coronary artery between Groups F-F and AF-F (both groups were classified as resistant to the initial IVIG) using Pearson’s chi-squared test for categorical variables and the Mann–Whitney U test for continuous variables. Additionally, we compared the clinical characteristics, blood laboratory findings before the initial IVIG treatment, and echocardiographic findings of the coronary artery between Groups AF-F and AF-AF (Group AF-AF was classified as responsive to the initial IVIG) using the same statistical methods. For the further comparison between Group F-F and Group AF-F, we added the information of white blood cell count, hemoglobin, albumin, and pyuria to the laboratory findings and evaluated each item.

## 3. Results

### 3.1. Clinical Characteristics of Enrolled Patients

A total of 306 patients were enrolled in this study (median age, 27 months [IQR, 15–44]; male, n = 173 [56.5%]). The median time for the initiation of the IVIG treatment and the achievement of treatment success were day 5 of illness (IQR, 4–6) and day 7 of illness (IQR, 6–8). Of the 40 patients who were febrile at the completion of the initial IVIG treatment, 38 remained febrile 24–36 h after completing the initial IVIG treatment. Among patients who were afebrile 24–36 h after completing the initial IVIG treatment, none of the patients in Group F-AF experienced a recurrence of fever, whereas 14 of 229 patients in Group AF-AF relapsed with fever and were introduced to the second IVIG treatment (Figure 1). Two hundred and seventeen patients (70.9%) were successfully treated with the initial IVIG treatment. All 89 patients (29.1%) with initial IVIG resistance received the second IVIG treatment as their second-line treatment, and 54 of them showed defervescence with the second IVIG treatment. The remaining 35 patients, in whom the second-line treatment failed and received infliximab, cyclosporine, or plasma exchange, were successfully treated with the 4th to 6th treatments. Fourteen patients (4.6%) developed CAAs immediately before the initial IVIG treatment, while seven patients (2.3%) developed CAAs at 4 weeks after the onset of KD. CAAs occurred within 4 weeks of the onset of KD in 24 patients (7.8%). The age, sex, day of illness at the initiation of the initial IVIG treatment, success rate of initial IVIG treatment, and incidence of CAAs in patients enrolled in this study were comparable to those of the Japan National Survey [24].

### 3.2. Comparison of the Clinical Characteristics, Laboratory Findings Before Initial IVIG Treatment, and Echocardiographic Findings of the Coronary Artery Between Group F-F and Group AF-F (Both Groups Showed Initial IVIG Resistance)

To determine whether the severity of acute KD can be predicted by body temperature just after completing the initial IVIG, we compared the clinical characteristics, including the incidence of CAAs, between Groups F-F (n = 38) and AF-F (n = 37) (Table 1). There were significant differences between the two groups in age (F-F vs. AF-F, 34.5 vs. 25.0 months, *p* = 0.046) and CRP (F-F vs. AF-F, 10.4 vs. 7.7 mg/dL, *p* = 0.021).

The median number of treatments needed for complete recovery was three in Group F-F and two in Group AF-F. This finding indicates that most patients in Group AF-F became afebrile after the second IVIG treatments; thus, few cases required a third IVIG. On the other hand, more than half of the patients in Group F-F remained febrile after the second IVIG, leading to a third-line treatment. From the perspective of the defervescence effect of IVIG treatment, Group F-F appeared more resistant to IVIG treatment. The incidence of CAAs was higher in Group F-F than in Group AF-F from just before the initial IVIG therapy to 4 weeks after the onset of KD (Table 1). Group F-F exhibited a higher incidence of CAAs from the disease onset. In summary, Groups F-F and AF-F were classified as resistant to initial IVIG; however, significant differences were observed between the groups in their response to additional treatment and in the incidence of CAAs.

### 3.3. Comparison of the Clinical Characteristics, Blood Laboratory Findings Before Initial IVIG Treatment, and Echocardiographic Findings of the Coronary Artery Between Groups That Were Afebrile at the Completion of Initial IVIG Treatment (Groups AF-F and AF-AF)

We compared the clinical characteristics of patients who were afebrile at the completion of the initial IVIG (Groups AF-F and AF-AF) (Table 2). There were significant differences between Groups AF-F and AF-AF in the neutrophil ratio (AF-F vs. AF-AF, 75.7% vs. 69.2%, *p* = 0.048) and AST (AF-F vs. AF-AF, 48U/L vs. 36 U/L, *p* = 0.022). The incidence of CAAs in Group AF-F, which was classified as resistant to initial IVIG treatment, was not significantly higher than that in Group AF-AF, which was classified as responsive to the initial IVIG treatment.

## 4. Discussion

Our study demonstrated that KD patients who remained febrile both at the completion of the initial IVIG treatment and 24–36 h later exhibited a high incidence of CAAs, whereas KD patients who became afebrile at the completion of the initial IVIG treatment exhibited a low incidence of CAAs. Therefore, we believe that the febrile status just after completing the initial IVIG treatment may serve as an early predictor of CAA incidence.

To our knowledge, one study reported that the fever pattern from the initiation of the initial IVIG treatment to 24 h after its completion was associated with the severity of KD [25]. Tanaka et al. reported that the incidence of CAAs was significantly higher in patients who experienced persistent fever up to 24 h after the completion of the initial IVIG treatment, relative to other patients (the incidence of CAAs was 27.3% in patients who experienced persistent fever from the initiation of the initial IVIG treatment to 24 h after its completion, whereas it was 0.0% in patients who experienced recurrent fever at 24 h after the completion of the initial IVIG treatment, and 6.8% in the patients who became afebrile at 24 h after the completion of the initial IVIG treatment, *p* = 0.0028) [25]. Our findings are similar to these data, except that they evaluated disease severity 24 h after the completion of the initial IVIG treatment, whereas we assessed it at the completion of the initial IVIG treatment, which was 24–36 h earlier than the previous study.

The advantage of an earlier evaluation is that the earlier introduction of second-line treatment, if needed, may help to minimize inflammatory damage to the coronary arteries. Histopathological studies of Japanese KD patients showed that, on days 6–8, the media of affected coronary arteries exhibit edematous dissociation of smooth muscle cells; on days 8–10, inflammatory cells infiltrate all layers of the arterial wall; and by days 10–12, the dilation of the affected coronary arteries begins [17,18]. Therefore, in the management of patients with acute KD, it is critically important to determine the severity of the disease as early as possible. However, earlier evaluations may lead to excessive second-line treatments. In our study, 38 (95%) of the 40 patients who were febrile at the completion of the initial IVIG treatment remained febrile at 24–36 h after completion. Therefore, it is important to explain to patients the rationale for the introduction of secondary IVIG based on earlier evaluation, which may not be necessary for approximately 5% of patients.

The present study had several limitations. The total duration of fever for each patient is indeed an important factor for CAL formation; however, we were not able to fully examine the relationship between total fever duration and CAL formation since this study focused on febrile status and subsequent IVIG treatments. This study was a single-center, retrospective cohort design consisting of only Japanese patients. A further prospective cohort study with a larger number of KD cases across multiple institutions is needed to understand the effects of not only treatment but also of contributing factors related to CAL formation.

## 5. Conclusions

In Japanese patients with acute KD, the febrile status just at the completion of the initial IVIG may serve as an early predictor of the occurrence of CAA. If Japanese patients with acute KD remain febrile at the completion of the initial IVIG treatment, the earlier initiation of a second-line treatment may help reduce the incidence of serious sequelae of CAAs. A multicenter cohort study is essential to determine the universal applicability of the findings from this preliminary study; therefore, we are planning to conduct such a study in Japan to validate these results.

## Figures and Tables

**Table 1:** Comparison of the clinical characteristics and laboratory findings before the initial IVIG treatment and echocardiographic findings of the coronary artery between Group F-F and Group AF-F.

	F-F (n = 38)	AF-F (n = 37)	*p* Value
	Median (IQR) or n (%)	Median (IQR) or n (%)	
Age, months	34.5 (22–56.3)	25 (11.5–40.5)	0.046
Boys, number	21 (55.3%)	18 (48.7%)	0.57
Day of illness at the initiation of initial IVIG	5 (4–5)	4 (4–5)	
Blood laboratory findings before initial IVIG			
WBC, /μL	18,100 (15,125–23,700)	15,700 (10,700–19,750)	0.003
Neutrophil ratio, %	81.1 (71.5–86.7)	75.7 (64.6–82.5)	0.18
Hemoglobin, g/dL	11.0 (10.1–11.5)	11.3 (10.2–12.0)	0.21
Platelet count, ×10^4^/L	26.7 (24.0–31.5)	26.8 (23.0–30.6)	0.13
CRP, mg/dL	10.4 (6.9–17.5)	7.7 (4.5–9.8)	0.021
AST, IU/L	54 (28–340)	48 (31–206)	0.80
Albumin, g/dL	2.9 (2.6–3.6)	3.4 (3.0–3.7)	0.008
Na, mmol/L	133 (132–135)	135 (134–137)	0.92
Pyuria, number	12 (31.6%)	13 (35.1%)	0.107
Received 2nd-line treatment, n	38 (100%)	37 (100%)	
The number of treatments needed for complete recovery, number	3 (2–3)	2 (2–2)	
Number of days until complete resolution of fever	9 (9–11)	8 (7–9)	0.005
Incidence of CAA, n			
Just before initial IVIG treatment	13 (34.2%)	0 (0.0%)	0.0053
One week after onset of KD	21 (55.3%)	0 (0.0%)	<0.0001
Two weeks after onset of KD	15 (39.5%)	0 (0.0%)	<0.0001
Four weeks after onset of KD	5 (13.2%)	0 (0.0%)	0.022
Within 4 weeks after onset of KD	21 (55.3%)	0 (0.0%)	<0.0001
Z score of coronary artery diameters			
Just before initial IVIG treatment			
RCA	1.59 (1.14–1.90)	0.94 (0.81–1.03)	<0.0001
LMT	1.69 (1.24–2.05)	1.02 (0.90–1.13)	<0.0001
LAD	1.61 (1.14–1.97)	0.96 (0.85–1.07)	<0.0001
One week after onset of KD			
RCA	1.85 (1.22–2.09)	0.96 (0.83–1.15)	<0.0001
LMT	2.03 (1.36–2.22)	1.05 (0.95–1.24)	<0.0001
LAD	1.87 (1.25–2.14)	1.02 (0.87–1.19)	<0.0001
Two weeks after onset of KD			
RCA	1.67 (1.08–1.93)	0.86 (0.77–1.03)	<0.0001
LMT	1.88 (1.19–2.09)	0.99 (0.89–1.10)	<0.0001
LAD	1.72 (1.13–2.02)	0.91 (0.81–1.05)	<0.0001
Four weeks after onset of KD			
RCA	1.39 (0.93–1.60)	0.78 (0.73–0.92)	<0.0001
LMT	1.58 (1.04–1.82)	0.90 (0.86–1.02)	<0.0001
LAD	1.45 (0.99–1.70)	0.84 (0.78–0.95)	<0.0001

**Table 2:** Comparison of the clinical characteristics and blood laboratory findings before the initial IVIG treatment and echocardiographic findings of the coronary artery between Group AF-F and Group AF-AF.

	AF-F (n = 37)	AF-AF (n = 229)	*p* Value
	Median (IQR) or n (%)	Median (IQR) or n (%)	
Age, months	25 (11.5–40.5)	26 (15–43.5)	0.71
Boys, number	18 (48.7%)	133 (58.1%)	0.28
Day of illness at the initiation of initial IVIG	4 (4–5)	5 (5–6)	
Blood laboratory findings before initial IVIG			
Neutrophil ratio, %	75.7 (64.6–82.5)	69.2 (59.1–79.3)	0.048
Platelet count, ×10^4^/L	26.8 (23.0–30.6)	29.3 (23.8–34.3)	0.14
CRP, mg/dL	7.7 (4.5–9.8)	6.8 (4.2–10.9)	0.96
AST, IU/L	48 (31–206)	36 (28–62)	0.041
Na, mmol/L	135 (134–137)	136 (134–137)	0.68
Received 2nd-line treatment, n	37 (100%)	14 (6.1%)	
The number of treatments needed for complete recovery, number	2 (2–2)	1 (1–1)	
Number of days until complete resolution of fever	8 (7–9)	6 (6–7)	<0.0001
Incidence of CAA, number			
Just before initial IVIG treatment	0 (0.0%)	1 (0.4%)	0.69
One week after onset of KD	0 (0.0%)	3 (1.3%)	0.48
Two weeks after onset of KD	0 (0.0%)	3 (1.3%)	0.48
Four weeks after onset of KD	0 (0.0%)	2 (0.9%)	0.57
Within 4 weeks after onset of KD	0 (0.0%)	3 (1.3%)	0.48
Z score of coronary artery diameters			
Just before initial IVIG treatment			
RCA	0.94 (0.81–1.03)	0.74 (0.71–0.81)	<0.0001
LMT	1.02 (0.90–1.13)	0.83 (0.79–0.89)	<0.0001
LAD	0.96 (0.85–1.07)	0.78 (0.74–0.85)	<0.0001
One week after onset of KD			
RCA	0.96 (0.83–1.15)	0.76 (0.71–0.83)	<0.0001
LMT	1.05 (0.95–1.24)	0.84 (0.79–0.92)	<0.0001
LAD	1.02 (0.87–1.19)	0.79 (0.74–0.88)	<0.0001
Two weeks after onset of KD			
RCA	0.86 (0.77–1.03)	0.73 (0.68–0.77)	<0.0001
LMT	0.99 (0.89–1.10)	0.82 (0.77–0.87)	<0.0001
LAD	0.91 (0.81–1.05)	0.77 (0.71–0.82)	<0.0001
Four weeks after onset of KD			
RCA	0.78 (0.73–0.92)	0.72 (0.68–0.76)	<0.0001
LMT	0.90 (0.86–1.02)	0.80 (0.76–0.84)	<0.0001
LAD	0.84 (0.78–0.95)	0.75 (0.71–0.80)	<0.0001

## Data Availability

The data presented in this study are available upon request from the corresponding author due to legal reasons.
